# Novel Approaches to Optimize Treatment Strategies in Glaucoma

**DOI:** 10.1155/2021/9876478

**Published:** 2021-08-16

**Authors:** Miriam Kolko, Steffen Heegaard, Barbara Cvenkel

**Affiliations:** ^1^Department of Drug Design and Pharmacology, University of Copenhagen, Copenhagen, Denmark; ^2^Department of Ophthalmology, Copenhagen University Hospital, Rigshospitalet-Glostrup, Denmark; ^3^Department of Pathology, Copenhagen University Hospital, Rigshospitalet-Glostrup, Denmark; ^4^Department of Ophthalmology, University Medical Centre Ljubljana, Ljubljana, Slovenia; ^5^Faculty of Medicine, University of Ljubljana, Ljubljana, Slovenia

Preserving lifelong vision in glaucoma patients with minimal impact on quality of life in terms of inconvenience and side effects is the goal of all treatment guidelines. The current understanding of glaucoma is limited, and only strategies that reduce intraocular pressure (IOP) have been shown to be effective in just delaying the progression of glaucoma. However, IOP-lowering treatments come with the risk of side effects that affect adherence and patients' quality of life. Often, treatments are initiated too late due to the asymptomatic nature of the disease, and progression to severe visual impairment or blindness is inevitable.

The current Special Issue addresses some aspects of basic and clinical research on neuroprotection, neurodegeneration, and medical treatment of glaucoma with an overview of the current status, including novel drugs and drug delivery systems to improve adherence as well as new approaches to potential treatment modalities in the future.

Glaucoma, like many chronic diseases, has a low adherence rate, which may lead to a progression of the disease and therefore to higher costs. Meier-Gibbons and Toteberg-Harms present an overview of the published literature on the costs of glaucoma care, dividing them into indirect, associated with the consequences of an advanced disease, and direct costs, associated with diagnostic tests and treatment. The published studies focus primarily on the direct cost of glaucoma care and show great differences in the price of antiglaucomatous drugs across countries and trends of prescribing generic drugs in Europe. Despite the fact that patients appear to be better informed about their disease and there has been an improvement in medical and surgical treatment, adherence has not improved in recent decades. More studies are needed to investigate the relationship between cost and adherence and focus on strategies for improving patients' adherence.

The use of generic medications for glaucoma has grown considerably in recent years. This is due to patent expiration dates for the innovators and the relative ease and short time to bring generic eye drops to the market, which does not require studies comparing branded and generic eye drops. With approval by the relevant regulatory authority, generics are required to have the same ingredient, route of administration, dosage, and be manufactured to the same quality standards as the reference medicinal product but may have different ingredients and packaging. Tatham reviews the potential pros and cons of generic drugs in terms of efficacy, cost, tolerability, and differences in formulation, adherence, and ease of use and perceptions of generic medicines among healthcare professionals and the general public. The switch to generic drugs reduces health-related costs, but there are some limitations of generic drugs that need to be addressed, such as the influence of inactive ingredients on bioavailability and thus efficacy and bottle design to improve consistency in drug delivery.

Long-term glaucoma treatment with multiple benzalkonium chloride- (BAK-) preserved eye drops may lead to increased aqueous humour flare. Pakuliene et al. used laser flare photometry, a noninvasive quantitative measurement of anterior chamber protein levels, to investigate intraocular inflammation in glaucoma patients on topical treatment for more than 2 years scheduled for cataract surgery without any other ocular abnormalities. The control group included cataract subjects without glaucoma. Investigators found higher aqueous humour flare in the glaucoma patients than in the control group. Several factors were associated with the aqueous humour flare increase, including pseudoexfoliation syndrome, number of eye drops, and presence of BAK.

An update of currently available IOP-lowering medications and potential new treatment targets for IOP-lowering and neuroprotective therapy are discussed by Cvenkel and Kolko along with the future trends in glaucoma therapy such as sustained drug delivery systems and drug formulations. For future personalized medicine based on genetic and other characteristics of the individual patients, it will be appropriate to identify patients with high risk of progression and treat them more vigorously.

In a review of the literature on vitamin D and glaucoma, Abouzeid and Samer concluded that vitamin D metabolites may play a role in glaucoma, either through a lowering effect on IOP or a neuroprotection pathway, but the exact molecular mechanism is not known. However, the limited number of clinical studies and significant bias in the study design preclude conclusions regarding the involvement of vitamin D in primary open-angle glaucoma (POAG).

Several mechanisms have been implicated in retinal ganglion cell death, and therapeutic strategies may be needed to target this to delay progressive retinal ganglion cell death in glaucoma. Mitochondrial dysfunction has been linked to optic neuropathy and brain neurodegenerative diseases. The review of Duarte focuses on how damaged mitochondria may impact the ability of neurons and glial cells to maintain homeostasis and induce sterile inflammation and neurodegeneration via mitochondrial damage-associated molecular patterns.

Tsai focuses on IOP-independent strategies for neuroprotection and/or neurodegeneration, including research into neurotrophic factors, gene therapy, immune system modulation, and novel neuroregeneration pathways. However, these innovative strategies should be critically balanced and weighed against the risk of disrupting the complex central nervous system environment.

Nuzzi et al. address the current new treatment strategies, including medical therapy and delivery modes, laser treatment, and minimally invasive glaucoma surgery. Furthermore, the most recent approaches to treat glaucoma in different stages are presented, including IOP-lowering effect of trans-palbebral electrostimulation of trabecular meshwork and novel techniques of cilioablation, as well as the advantages and risks of stem cell therapy, neurotrophic factors and potential therapy with stem cell-derived exosomes.

An important research goal is identifying systemic risk factors that can help in predicting POAG. Pfahler et al. using nailfold capillaroscopy documented increased levels of nailfold haemorrhage, dilated capillaries, and avascular zones in POAG patients. These findings suggest that systemic microvascular dysfunction is frequent in POAG. In addition, the presence of any haemorrhage was found to be a highly significant risk factor for glaucoma.

An enhanced physiological stress response to reduced oxygen supply has been documented in patients with normal tension glaucoma (NTG) compared to age-matched healthy controls, supporting the role of vascular dysfunction. Dalgaard et al. measured serum adrenaline and endothelin-1 levels and changes in distal finger temperature. In patients with NTG, a relative increase in adrenaline was found during hypoxia and a relative decrease during recovery, but not in controls. Hypoxia also induced elevated temperature in distal finger in patients with NTG only.

The role and function of synucleins, a family of small proteins involved in neurodegeneration of the central nervous system, have been studied in an animal model of glaucoma. Liu et al. found that IOP elevation caused a significant loss of retinal ganglion cells in older animals, but no significant change in young rats. Beta-synuclein was significantly downregulated in young animals without any significant change in old ones. These results show a link between aging and beta-synuclein regulation. Hydrogen sulfide, a potent reductant, was effective in downregulating beta-synuclein and was neuroprotective against acute IOP elevation.

Finally, Russel et al. devised a low-cost method assessing glaucoma using digital image analysis of the angle and optic nerve. Colour fundus photographs, standard optic disc OCT, and digital slit lamp gonioscopy images were used for digital image conversion and analysis. Requiring only retina digital cameras, the volumetric, geometric, and segmentational data acquired through digital image analysis correspond well with the data obtained by OCT imaging, but importantly reduce the costs.

In summary, this special issue offers an overview of novel treatment strategies and future targets in the treatment of glaucoma. It also provides new findings that may be a clue for new research in this important field.

